# Running an inclusive and accessible teleophthalmology service for people with disabilities

**Published:** 2022-06-07

**Authors:** Kriti Shukla

**Affiliations:** 1 Research Associate: Centre for Health Outcome Research and Economics, Indian Institute of Public Health, Hyderabad, India.

**People with disabilities face various barriers when making use of teleophthalmology, and these must be addressed with care when planning and implementing services.**

**Figure:**
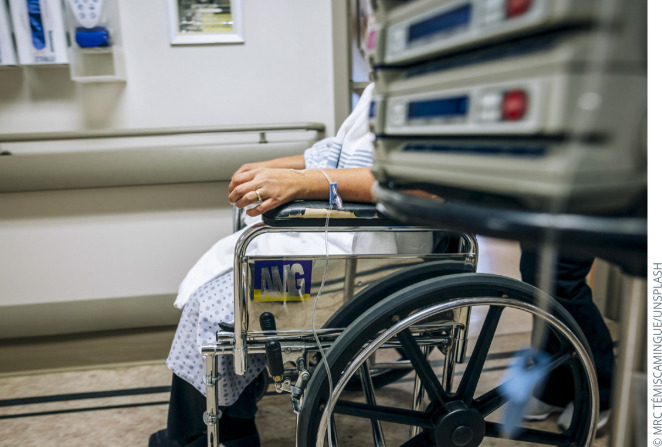
Eye care centres that offer teleophthalmology services must be physically accessible and user friendly. **CANADA**

The ongoing pandemic has seen an increased push to improve remote access to health care, including eye care. However, despite the advances in telehealth and teleophthalmology, people with disabilities continue to face varied and complex challenges when accessing health services. The level of challenge people face depends on the type and severity of impairment and their age, gender, and socioeconomic status. In addition, there are the ‘digital determinants of health’^1^: the degree of access people have to the internet and to assistive and communication devices, and their level of digital literacy.

The 184 countries that ratified Article 25 of the United Nations Convention on the Rights of Persons with Disabilities (UNCRPD) are legally bound to ensure that persons with disabilities enjoy the highest attainable standard of health without facing discrimination based on disability.^2^ This also implies that health care providers have the responsibility to ensure that services such as teleophthalmology are inclusive for people with disabilities.

## Adapting teleophthalmology for people with disabilities

Teleophthalmology can allow people with disabilities to receive high quality and affordable eye care from the comfort of their home or at an easy distance from it. This can help them avoid the barriers of travel costs, travel and waiting time, and the need for support from a caregiver. Importantly, however, teleophthalmology services must themselves also be accessible and disability-friendly and not create new barriers.

Equally, it is important that people with disabilities have adequate representation in the design of telehealth services at the regional, national, and policy levels. It would also be good practice to seek their help to give feedback to ensure services are user friendly.

**People with visual impairment or blindness** use screen readers (such as NVDA, JAWS, or VoiceOver) to read what is on their screen. The teleophthalmology platform should therefore be compatible with assistive technology and Braille keyboards. Screen readers cannot read scanned documents, images, or infographics if no alternative text is provided. It is, therefore, recommended that optical character recognition (OCR) technology is used to scan physical documents (records, lab notes, reports, and prescriptions) before uploading them to a digital or telemedicine platform. The OCR software converts scanned documents to readable text documents. In addition, colour contrast and text magnification options are crucial for allowing people with low vision to access the platform.

Patients need clear instruction, in accessible formats, about how to upload photos of the eye. Tools such as VoiceOver in iOS devices make the camera app accessible to people with blindness. If such technology is unavailable, a friend or caregiver's help may be needed to take the picture.

**People with mobility impairments** may find it difficult to do tasks requiring fine motor skills, such as controlling the mouse to move the cursor or clicking multiple times to navigate a site. They may also find it challenging to set up appointments if they have to complete long or complicated forms. Well-organised information and forms with fewer fields can enhance access.

Depending on their level of impairment, **people with** **speech and hearing impairments** might use alternative communication devices or sign language interpretation to communicate with the eye care provider. Therefore, the teleconsultation platform should ideally make it possible to include an online interpreter on the same video call with the patient and eye care provider.

A possible solution in a low-resource setting would be to recruit and train several freelance interpreters so that one of them will be able to join in the audio or video call. Allied ophthalmic personnel can provide two to three days of training to interpreters to explain the medical terminology commonly used in eye care; this will avoid misunderstandings or losing crucial information while interpreting. In India, the Indian Sign Language Research and Training Centre (ISLRTC) maintains a directory of sign language interpreters.^3^ Such directories serve as a good resource for interpretation services as and when required.

Although masks are indispensable in pandemic times, an ophthalmologist undertaking an online consultation with a **patient with hearing impairment** could use a transparent face shield instead. This small change would help those using cochlear implants or hearing aids to lip read and see expressions, which helps them to follow the conversation. The chat option may also be helpful when communicating in a low-resource setting.

It is also vital to provide adequate captioning in regional languages for video content or educational/awareness materials that health providers put online so that these are accessible to everyone.

**People with intellectual or developmental disabilities** may not be comfortable explaining their eye health problems over the phone or video chat. Caregivers can be included in the conversation after taking consent from the patient with a disability.

Many people with disabilities, and older people in low-, and middle-income countries, are likely to have lower socioeconomic status, low levels of digital literacy, and limited or no access to the internet or smartphones; thus, they are unable to benefit from teleconsultations. To reach out to such a population, an eye hospital may have to arrange transport – with the help of non-governmental organisations (NGOs) or disabled people's organisations (DPOs) – that would allow people to visit the nearest clinic or vision centre where teleophthalmology services have been established.

**Table 1:** How to make teleophthalmology services accessible for people with disabilities

**Barriers faced**	**Action suggested**
**Inaccessible teleconsultation platform**	Ensure that your teleconsultation platform complies with accessibility standards and guidelines. In case your government has not yet adopted such guidelines, refer to the Web Content Accessibility Guidelines online (WCAG)^4^ Involve disabled people's organisations (DPOs) in the planning stages and get your platform audited for accessibility
**Communication barriers**	Capture patients’ accessibility needs and support requirements when appointments are made Give disabled patients flexible and longer appointment times to avoid rushing Check what type of disability a patient has and make arrangements accordingly (e.g., arrange a sign language interpreter, or use audio messages) When speaking to a person with a visual disability, identify yourself and explain your role (e.g., “Hello, I am …, and I am here to help you with …”) Listen attentively to the person with speech impairment; let them complete the sentence at their pace and allow adequate time for them to ask questions Provide prescriptions in a preferred format such as Braille, large print, or audio messages Use optical character recognition (OCR) technology to scan documents before uploading them to a website and make the text-based version available too Provide adequate captioning in regional language for the website's audio visual content
**Low digital literacy or lack of access to digital devices and the internet**	Provide home visits by eye care workers for patients who do not have active internet or devices Provide a toll-free helpline number for patients to contact the service they need Arrange transport with the help of NGOs or DPOs to the nearest primary eye care centre

In the absence of such a centre, or when travel restrictions are imposed during a pandemic, a vision guardian or health worker – who may have a mobile internet device – can identify patients who require an eye care consultation and visit them at home. Providing a toll-free helpline number (which people can call to speak to a clinician) can also be an option.

If teleophthalmology services are planned at an eye care centre, the facility must be physically accessible and user friendly, equipped with universal design elements such as accessible parking, signage, step-free entrances, ramps, lifts, accessible toilets, tactile tiles, grab bars, and accessible examination spaces and diagnostic equipment.

Eye care service providers should work closely with the manufacturers of telehealth platforms to ensure that the needs of people with disabilities are anticipated and planned for from the start. Standardisation can address most of these barriers. For the latest guidelines on ICT accessibility, please see Web Content Accessibility Guidelines (WCAG)4 at https://bit.ly/3LfX8dN.

The most important thing to remember when providing services for people with disabilities is to make contact with them in advance to understand their preferred mode of communication. Table 1 lists the barriers typically faced by people with disabilities in accessing remote eye health services and some ways to overcome them.

Disability sensitisation workshops for eye care workers can also be planned in liaison with DPOs to improve staff members’ understanding of the barriers faced by people with disabilities in accessing eye care services. Developing a protocol for teleconsultations, and training staff members who provide teleconsultation services, will make this more comfortable for staff members and the patients who seek eye care.
